# Fission of Tubular Endosomes Triggers Endosomal Acidification and Movement

**DOI:** 10.1371/journal.pone.0019764

**Published:** 2011-05-10

**Authors:** Kumi Mesaki, Kenji Tanabe, Masanori Obayashi, Natsuko Oe, Kohji Takei

**Affiliations:** Department of Neuroscience, Okayama University Graduate School of Medicine, Dentistry and Pharmaceutical Sciences, Okayama, Japan; University of Nebraska Medical Center, United States of America

## Abstract

The early endosome acts as a sorting station for internalized molecules destined for recycling or degradation. While recycled molecules are sorted and delivered to tubular endosomes, residual compartments containing molecules to be degraded undergo “maturation” before final degradation in the lysosome. This maturation involves acidification, microtubule-dependent motility, and perinuclear localization. It is currently unknown how sorting and the processes of maturation cooperate with each other. Here, we show that fission of a tubular endosome triggers the maturation of the residual endosome, leading to degradation. Use of the dynamin inhibitor dynasore to block tubular endosome fission inhibited acidification, endosomal motility along microtubules, perinuclear localization, and degradation. However, tubular endosome fission was not affected by inhibiting endosomal acidification or by depolymerizing the microtubules. These results demonstrate that the fission of recycling tubules is the first important step in endosomal maturation and degradation in the lysosome. We believe this to be the first evidence of a cascade from sorting to degradation.

## Introduction

Degradative pathways play an important role in signal termination, antigen presentation, and digestion of unnecessary materials by lysosomal degradation [1], [2]. These pathways are distinct from recycling pathways, which recycle internalized molecules to the cell surface [3], [4]. Although the two pathway types differ from each other, cargos for either pathway are primarily delivered to and separated at the same small, peripherally-located, tubulo-vesicular, early endosomes [5], [6]. The early endosome displays lateral heterogeneity in receptor distribution, suggesting that the existence of barriers to receptor diffusion at the junction of tubules and vesicles [7]. This heterogeneity is achieved, in part, by selecting cargos destined for degradation. Cargos for degradation typically contain a sorting signal such as ubiquitin, and are positively sorted to and accumulate in the vacuolar part of the endosome. Unselected residual cargos for recycling are negatively sorted into the tubular microdomain. These tubules are then severed from the endosome and recycled to the cell surface [7], [8], [9], [10], [11].

After this fission process, early endosomes containing cargos destined for degradation undergo intraluminal acidification [by proton-pumping vacuolar-ATPase (V-ATPase)], move along microtubules, and are localized to the perinuclear region before being degraded [12], [13]. This maturation process has been first proposed by Helenius and Mellman (1983) and supported by series of subsequent papers (reviewed in Mellman, 1996). Although it is suggested that recycling components were rapidly removed prior to the transition from early to late endosomes [14], it remains unclear whether these events take place independently or influence one another.

To determine the significance of and the relationship between each step of the degradative pathway, researchers have performed various inhibition studies. For instance, microtubule disruption by nocodazole inhibits the motility of endosomes and prevents degradation [13], [15]. Inhibition of dynein, a minus-end–directed motor protein, results in retarded degradation [16], while overexpression or depletion of KIF16B, a plus-end–directed kinesin motor that transports early endosomes in a Rab5- and PI3K-dependent manner, disrupts endosomal distribution [17]. Conversely, blocking endosomal acidification by the V-ATPase inhibitor bafilomycin A1 inhibits the degradative pathway from the early to late endosomal stages [15], [18]. Inhibition of tubule fission has not been reported, since no mechanism for its inhibition is currently known.

We sought to identify an inhibitor of tubule fission, and to use this inhibitor to define the significance of tubule fission on endosomal maturation. The specific dynamin inhibitor dynasore blocked tubule fission from early endosomes. Interestingly, dynasore inhibited both the recycling and degradative pathways of endosomal transport. By inhibiting tubule fission, we were able to conclude that the degradative pathway can be thought of as a cascade composed of five events, with tubule fission positioned at the top and functioning as a key regulator. Since this intracellular transport is essential for infection, which influences the immune system and the effects of toxins, we believe that the novel findings presented here will be useful for understanding of the effects of various drugs.

## Results and Discussion

### Recycled molecules are removed via tubular endosomes

**Figure 1:**
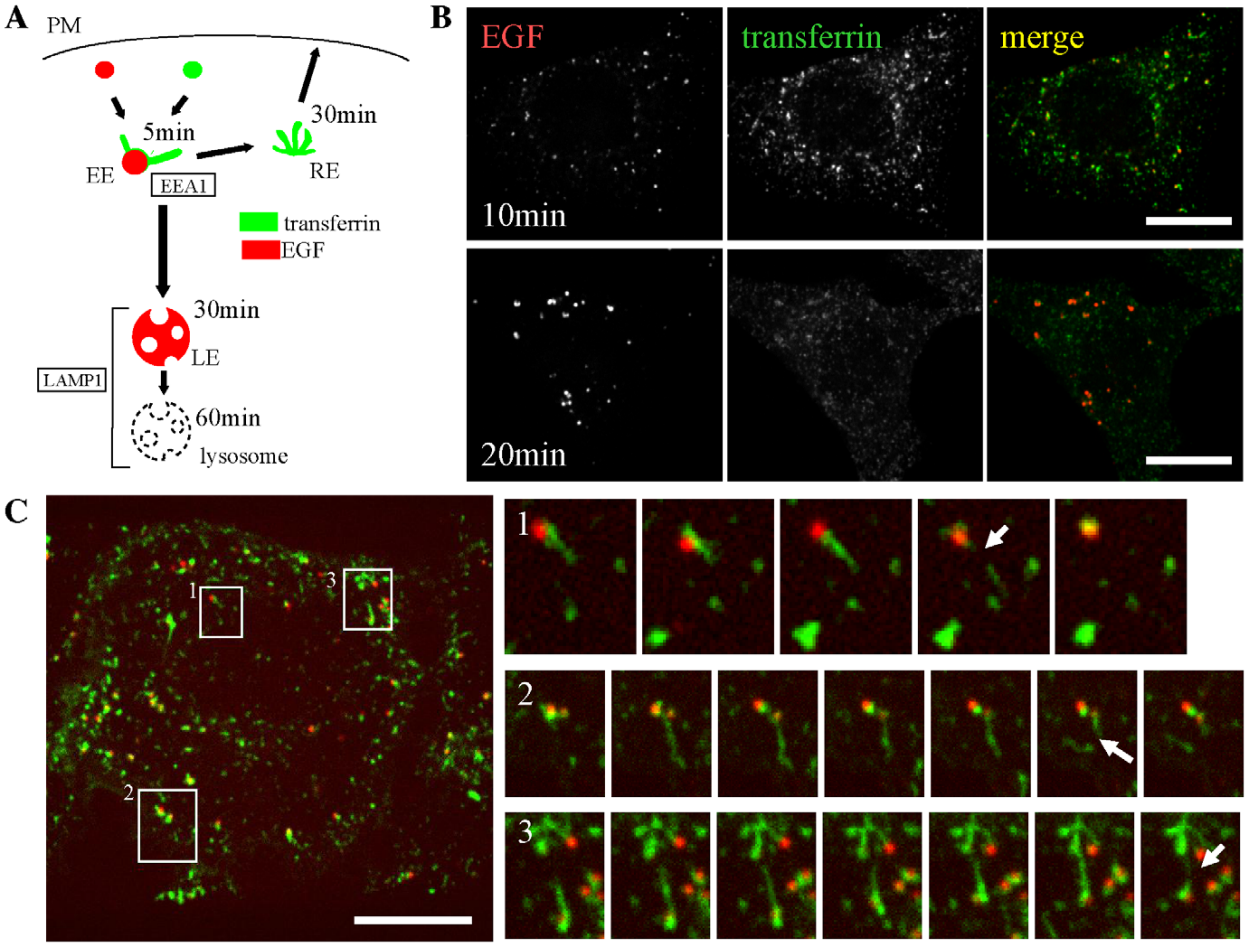
Recycling and degradative pathways are segregated by tubule fission. **A**, Recycling and degradative pathway are illustrated, with the typical time course of intracellular transferrin and EGF in our experiments shown. Boxed words represent the marker for each compartment. EE, early endosome; LE, late endosome; RE, recycling endosome. **B**, HeLa cells were bound to Alexa555-EGF and Alexa488-transferrin on ice. After rinsing, the cells were incubated at 37°C for the time indicated and fixed. Scale bar, 20 µm. **C**, Cells were bound with ligands and transferred to 37°C as above. Live images were taken 10 min post-internalization. Frames were captured every 2 sec. Scale bar, 20 µm. (Also see **Movie S1**)

Intracellular transport of internalized Alexa555-conjugated epidermal growth factor (EGF) and Alexa488-conjugated transferrin were used to visualize the degradative and recycling pathways, respectively. At 10 min post-internalization, both EGF and transferrin were transported to the same, relatively small, early endosomes located in the cell periphery (Figs. 1A and 1B). However, at 20 min post-internalization, EGF-positive endosomes were primarily localized to the perinuclear region and had enlarged, implying that some of these structures were actually multi-vesicular bodies. In contrast, most of transferrin had disappeared, suggesting that it was recycled back to the cell surface.

To observe the segregation of the two pathways in the early endosome, we performed live-cell imaging. Tubules containing transferrin radiated from the EGF-positive vacuolar portion. These tubules were motile and underwent fission at the tubule root, followed by segregation of EGF and transferrin (Fig. 1C and Movie S1). These observations are consistent with the following pathway model: degradative and recycling molecules are first transported to the same early endosome at the cell peripheral region. The degradative molecules are then positively selected to segregate to the vacuolar domain, while recycled molecules enter the tubular domain. Next, the tubular domain is segregated from vacuolar domain and is recycled back to plasma membrane via recycling endosomes [12], [13].

### Dynasore blocks the recycling pathway of the early endosome

We next sought to determine how tubule fission could be inhibited. It was previously predicted that the mechanochemical enzyme dynamin, a 100 kDa GTPase that pinches vesicles off during endocytosis, may play a similar key role during tubule fission [19], [20], [21], [22]. The role of dynamin in tubule fission has been investigated using dominant negative mutants and reduced endogenous expression [23], [24], but the relatively long time required between expression/reduction and observation made it difficult to investigate its effect on intracellular transport. Using a temperature-sensitive mutant, another study found that dynamin did not function in the early endosome, but rather in vesicle formation in the recycling endosome [25]. However, it is possible that the time required for the temperature-sensitive mutant to express its inhibitory effect was also too long for observation of any effect in the early endosome.

**Figure 2:**
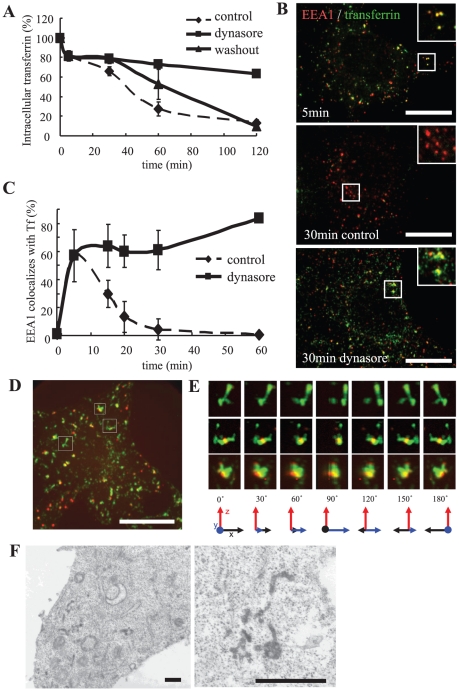
Dynasore inhibits transferrin recycling in early endosomes. **A**, Cells were incubated with biotin-conjugated transferrin on ice for 1 h, rinsed, and transferred to 37°C. At each time point, cells were lysed and intracellular transferrin was measured by ELISA. In the ELISA assay, the cell lysates bound to plates coated with goat anti-transferrin antibodies (EY Laboratories, San Mateo, CA) and were detected with streptavidin-HRP. **B**, HeLa cells were incubated with Alexa 488-transferrin (Molecular Probes) on ice for 1 h, and then processed as in **A**. After 5 min, the media was changed to medium containing DMSO (control) or dynasore. At the time indicated, the cells were fixed and stained for EEA1 (BD Transduction Laboratories, San Jose, CA). Scale bar, 20 µm. **C**, Colocalization of EEA1 and transferrin was measured and is represented mean ± S.D. N = 20 cells. **D**, HeLa cells were bound with Alexa 555-EGF (Molecular Probes) and Alexa 488-transferrin on ice for 1 h. After rinsing, the cells were incubated at 37°C for 5 min and dynasore was added. Thirty minutes after internalization, the cells were fixed and observed. Scale bar, 20 µm. Pictures were processed for 3D reconstruction using MetaMorph software (**E**). **F**, Cells were bound to HRP-transferrin and treated for 30 min as in **D**. The cells were then incubated in a DAB-containing solution on ice for 30 min, fixed, and processed for electron microscopy. Scale bar, 500 nm.

Recently, an inhibitor of dynamin, dynasore, was identified [26]. Taking advantage of its rapid action, we observed the effect of dynasore on tubule fission from the early endosome. In our experiments, an exposure time of 1 min was sufficient for dynasore to act, judging by endocytotic inhibition (data not shown). This concentration was higher than that of original report [26], probably due to presence of serum [27]. However, we didn't observe complete inhibition of dynamin-dependent endocytosis at lower concentration in HeLa, Cos-7 or U2OS cells (unpublished observation). To examine its effect on tubule fission from early endosomes, we added dynasore to cells at 5 min post-internalization, a time point when transferrin was mainly localized in the early endosome. Using ELISA, we found that a large part of the internalized transferrin (63.2%) remained in the dynasore-treated cells even after 120 min, by which point almost all of the internalized transferrin was recycled; only 12.8% remained in the control cells (Fig. 2A). This suggested that dynasore blocked intracellular transferrin transport somewhere along its way to the cell surface. When dynasore was removed, transferrin recycling proceeded similar to that in control, reflecting the reversibility of the inhibitor (Fig. 2A).

We performed immunofluorescence to determine at which step the recycling of transferrin was blocked in dynasore-treated cells. After its internalization, transferrin is transported to early endosomes that localize at the cell periphery. It is then sorted and transported primarily to recycling endosomes via tubules and then to the cell surface [3], [4], [28], [29]. Consistent with this, in control cells transferrin colocalized with EEA1 at 5 min post-internalization, but was separated from EEA1 after 30 min. In dynasore-treated cells, however, transferrin continued to colocalize with EEA1, indicating that it remained in the early endosome (Fig. 2B). Quantification of this colocalization revealed this phenotype clearly (Fig. 2C); 83.6% of the EEA1 signal still colocalized with transferrin after 60 min in dynasore-treated cells compared to only 0.6% in control cells. These results demonstrate that, in the recycling pathway, the export of transferrin from the early endosome was inhibited by dynasore.

### Treatment with dynasore induces tubular extension of early endosomes

To identify at which stage dynasore halted transferrin transport in the early endosome, we determined the structure of and domain formation by these endosomes. In dynasore-treated cells, transferrin-containing tubules were more frequently observed than control cells (Fig. 2D). Treatment with acid buffer (0.5M glycine, pH 2.2) had no effect on these signals (data not shown), suggesting that these tubular structures were not from plasma membrane. Others have reported the same observation [30]. Whereas EGF localized in vacuolar domains similar to control cells (Fig. 2D), EGF-positive compartments did not separate from transferrin-containing tubules by 30 min (Fig. 2E and Movie S2). EGF and transferrin appeared to be located in adjacent compartments. Closer observation with electron microscopy confirmed that there were many tubules in dynasore-treated cells (Fig. 2F). Based on these results, we hypothesize that dynamin plays a similar critical role in tubule fission from the early endosome as it does in vesicle formation. Therefore, when dynasore was added, transferrin tubules could be formed but could not undergo fission. They therefore froze just before fission and this resulted in the impairment of recycling.

It was possible that the same results would be obtained when blocking vesicular transport from the early endosome to the plasma membrane, which is a direct and rapid recycling pathway. We therefore tested this possibility by using LY294002, which is known to block only the direct recycling pathway from early endosomes via vesicles as a consequence of PI3K inhibition [31]. When this agent was used, the segregation of transferrin from EGF was largely unaffected (data not shown). Therefore, the impairment of recycling in dynasore-treated cells was presumed to involve the inhibition of tubule fission.

In addition to the observations above, we also observed morphological changes in the cell using electron microscopy. Under the influence of dynasore, mitochondria were swelled and enlarged (Figure S1). This observation seemed to reflect the inhibition of Drp1, a member of the dynamin superfamily that functions similar to dynamin during mitochondrial division [32]. This was logical, as dynasore inhibits the GTPase activity of both dynamin and Drp1 *in vitro* [26].

### Tubular endosomes fail to undergo fission from early endosomes following treatment with dynasore

**Figure 3:**
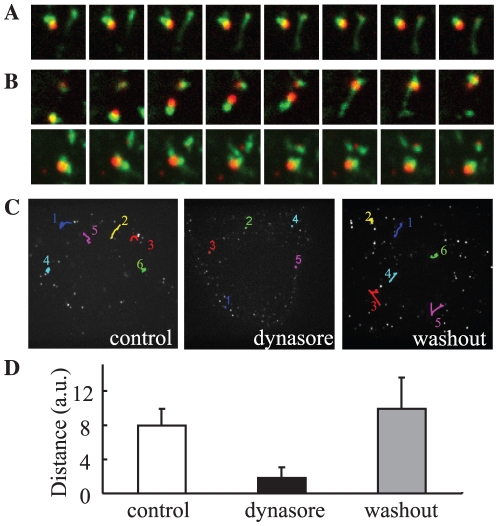
Dynasore inhibits tubule fission and endosomal motility. HeLa cells were bound on ice with Alexa555-EGF and Alexa488-transferrin, rinsed, and transferred to 37°C. After 5 min, dynasore was added, and the cells were incubated further for another 25 min. For washout, the cells were rinsed at 30 min with dynasore-free media. Live images were taken just before washout (**A**) and at 10 min after washout (**B**). Frames were captured every 2 sec for a total of 2 min 30 s (See also **Movie S3**). **C**, Movements of EGF-positive endosomes for 90 s were manually tracked and are illustrated. **D**, The mean total movement distance of EGF-positive endosomes for 2 min. 20 endosomes were measured from 4 cells; Error bar, S.D.

To validate our hypothesis that tubule fission was inhibited by dynasore, we performed live-cell imaging. In control cells, transferrin-containing tubules pinched off at the root and separated from the EGF-positive vacuolar part. As expected, when cells were treated with dynasore, transferrin tubules formed but fission was not observed (Fig. 3A and Movie S3). When dynasore was washed out, fission events were restarted and tubular transport of transferrin was observed (Fig. 3B and Movie S4). From these results, we concluded that dynasore inhibited tubule fission from the early endosome and resulted in inhibition of recycling.

### Movement of EGF-containing endosomes ceases following treatment with dynasore

**Figure 4:**
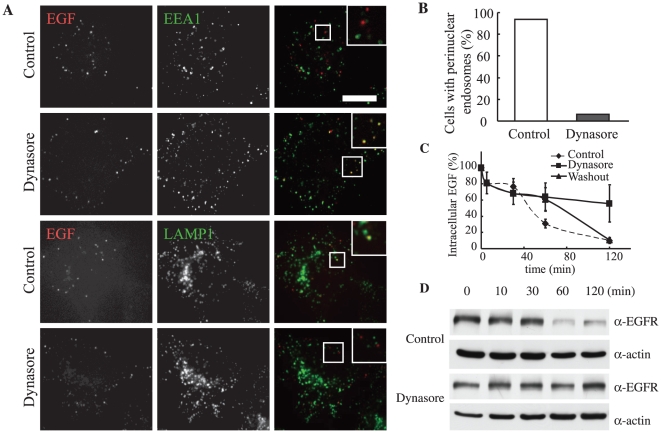
Degradative pathway is inhibited in the early endosome. **A**, HeLa cells were incubated with Alexa555-EGF on ice for 1 h, rinsed, and transferred to 37°C. After 5 min, the media was changed to DMSO- (control) or dynasore-containing media and incubated further for the time period indicated. Cells were fixed and stained using anti-EEA1 or anti-LAMP1 (Santa Cruz Biotechnology, Santa Cruz, CA) antibody. **B**, The cells displaying localized endosomes were manually counted. N = 80 cells. **C**, Cells were incubated with biotin-conjugated EGF on ice for 1 h and processed as in **Figure 1A**. Intracellular EGF was measured by ELISA. D, Cells were stimulated by EGF. At the indicated time after stimulation, cells were lysed and subjected to SDS-PAGE and an immunoblot analysis using an anti-EGF receptor or anti-ß-actin.

Interestingly, we noted a change in EGF localization and motility following treatment with dynasore with live-cell imaging. As mentioned above, EGF-containing endosomes moved toward the perinuclear region prior to lysosomal degradation. This juxtanuclear recruitment can be achieved by the microtubule-dependent endosomal motility. As shown in Fig. 3C, endosomes showed bidirectional short-range movement along the microtubule, which completely resulted in translocation around the nucleus. In the presence of dynasore, however, most of the motility disappeared (Fig. 3C and D). Although EGF-containing endosomes reached the perinuclear region in control cells, in the presence of dynasore they localized at the cell periphery without localizing around the nucleus. As summarized in Fig. 4B, endosomes were found around the nucleus in 94% of control cells compared to 6% of dynasore-treated cells. After washing out the dynasore, EGF-containing endosomes began to move, resulting in pericentriolar localization. A possible explanation for this phenomenon will be discussed later in the paper.

### Dynasore inhibits EGF transport from the early endosome

We have demonstrated that treatment of cells with dynasore resulted in the inhibition of tubular endosome fission and of the perinuclear localization of EGF-containing endosomes. The next question we asked was which steps in the degradative pathway proceeded without tubule fission. At 30 min post-internalization in control cells, EGF colocalized not only with EEA1 but also with LAMP1, a marker for late endosomes/lysosomes (Fig. 4A, Control). These EGF-containing endosomes were enlarged and located at the perinuclear region (Fig. 1B). Under the effect of dynasore, EGF colocalized with EEA1 and not with LAMP1 (Fig. 4A). This indicated that EGF remained in the early endosome, and the EGF compartment did not mature to the late endosome stage. The same results were obtained using exogenous markers such as Rab GTPases (Figure S2). The size of EGF-containing endosomes also indicated impairment in maturation, as these endosomes were enlarged in control cells but not in dynasore-treated cells (Fig. 4A). EGF degradation was inhibited with dynasore treatment, but restarted after dynasore washout. At 120 min post-internalization, significantly less internalized EGF remained in control cells (9.93%) compared to dynasore-treated cells (55.4%; reduced to 10.6% at 90 min after washout) (Fig. 4C). Further, EGFR degradation was inhibited in dynasore-treated cells (Fig. 4D). Taken together, these results indicate that the maturation of early endosomes into late endosomes, which is essential for degradation, was not achieved without fission of the recycling tubules in early endosomes.

### Fission loss leads to maturation defects due to impairment of endosomal acidification

We hypothesized that one possible explanation for why maturation did not occur without tubule fission was the inability of the endosomes to acidify. Acidification is performed by the proton pump V-ATPase that localizes to the vacuolar microdomain and to vacuoles heading to degradation [12]. Acidification of the intraluminal space of an endosome is essential for its maturation; the proteins required for maturation are recruited to the endosome following its acidification [33], [34], [35].

**Figure 5:**
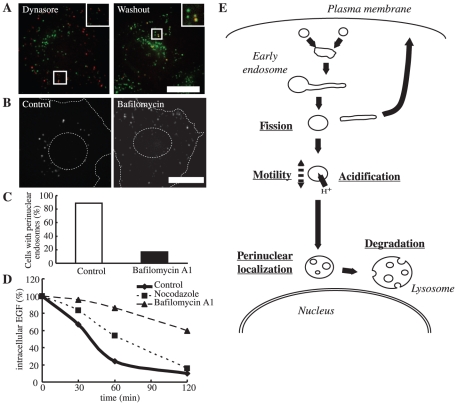
Acidification is required for perinuclear localization of endosomes, and a cascade model of the degradative pathway. **A**, HeLa cells were bound on ice with Alexa555-EGF, rinsed, and transferred to 37°C. After 5 min, dynasore was added. For washout, after 30 min the cells were rinsed and incubated in dynasore-free media. Images are just before washout (left) and 10 min after washout (right). **B**, HeLa cells were incubated with Alexa555-EGF on ice for 1 h, rinsed, and transferred to 37°C. After 5 min of internalization, DMSO (left) or Baf (right) was added and the cells were further incubated. After 30 min, the cells were fixed. Dashed-line circle, nucleus; Dashed-line (not circle), outline of the cell; Scale bar, 20 µm. **C**, The endosomes displaying perinuclear localization were manually counted. N = 80 cells. **D**, Cells were incubated with biotin-conjugated EGF on ice for 1 h. After rinsing, the cells were transferred to 37°C, incubated for the time indicated, and processed by ELISA. Nocodazole was added at 1 hour before the incubation, while bafilomycin A1 was added at 5 min after internalization. **E**, Both internalized EGF and transferrin initially enter the same early endosomes. EGF is sorted into vacuolar domains and unsorted transferrin is collected into tubules. After tubule fission, intraluminal acidification of EGF-containing endosomes proceeds, as endosomes are motile. As a result, enlarged endosomes are recruited around the nucleus and degradation is finally completed. Dynasore inhibits tubule fission, which blocks the subsequent steps. Bafilomycin A1 inhibits endosomal acidification and causes inhibition of perinuclear localization and degradation, but not of fission or motility. Nocodazole inhibits endosomal motility, resulting in impaired perinuclear recruitment and degradation, but normal endosomal acidification.

We hypothesized that dynasore causes the intra-endosomal spaces of tubule-associated early endosomes to become too large for normal acidification. This would lead to an inability of the early endosome to be adequately acidified for maturation. To confirm this, we investigated whether the EGF-positive compartment was acidified in dynasore-treated cells. Epidermal growth factor–containing endosomes were labeled at 30 min post-internalization with lysotracker, which detects acidic compartments. In control cells, endosomes were clearly labeled, indicating acidification of the endosomes (data not shown). As expected, EGF-containing endosomes in dynasore-treated cells did not label with lysotracker, but did label after dynasore was washed out (Fig. 5A). This suggests that there was insufficient early endosomal intraluminal acidification when the cells were treated with dynasore. However, after washout, sufficient acidification was achieved, as tubule fission caused a decrease in the volume of the intraluminal space.

### Endosomal acidification and motility along microtubules occur independently, but both are necessary for perinuclear localization

The above results demonstrate that in the absence of tubule fission, endosomal acidification, motility, and perinuclear recruitment were inhibited. We next asked whether there was any relationship between these three phenomena. Previous studies have shown that the disappearance of minus-end–directed motility with inhibition of dynein, a motor which moves along the microtubule toward the minus-end, results in a lack of juxtanuclear localization and impairment of maturation and degradation [16]. It has also been reported that depletion of KIF16B, a motor that moves along the microtubule towards the plus end, results in accumulation of the early endosomes in the perinuclear region and in enhanced degradation. KIF16B overexpression blocks the juxtanuclear localization of endosomes, leading to degradative inhibition [17]. Despite these results, the significance of acidification in the motility of endosomes remained unclear.

We determined the distribution and the motility of EGF-containing endosomes in the presence of bafilomycin A1 (Baf), a V-ATPase inhibitor that impairs endosomal acidification. In Baf-treated cells, EGF-positive endosomes showed no perinuclear localization and remained as small as those in dynasore-treated cells (Fig. 5B). We also noted degradative inhibition (Fig. 5D) similar to previous reports [15], [18]. Whereas, the segregation between degradative and recycling pathway was not affected by Baf treatment (see Figure S3). Interestingly, although observations of Baf-treated cells indicated that EGF-containing endosomes were mobile, their recruitment to the juxtanuclear region was not observed (Fig. 5C and Movie S5). This result was different from the frozen movement observed in dynasore-treated cells (Movie S3 and S5). To inhibit endosomal motility, we treated cells with nocodazole, an inhibitor of microtubule polymerization. In the presence of nocodazole, endosomal motility but not acidification disappeared, and degradation was retarded (Fig. 5D).

Taken together, our results demonstrated that when acidification was impaired (Baf treatment), perinuclear localization but not endosomal motility was inhibited; whereas when motility was impaired (nocodazole treatment), perinuclear localization but not endosomal acidification was inhibited. Consequently, we concluded that endosomal acidification and motility lie in parallel, and both are essential for endosomes to localize around the nucleus.

### Effects of the inhibitions of tubule fission, endosomal acidification, and motility

Our results indicated that tubule fission, endosomal acidification/motility along the microtubule, and perinuclear localization, which happen between the early endosomal state and degradation, took place in a defined order and could be precisely regulated (Fig. 5E and Table 1).

**Table 1:** Effects of drugs on degradative pathway.

Inhibitor	Tubule fission	Motility	Acidification	Prinuclear localization	Degradation
dynasore	No	No	No	No	No
nocodazole	Yes	No	Yes	No	No
bafilomycin A1	Yes	Yes	No	No	No

When tubule fission from the early endosome was halted by dynasore treatment, endosomal acidification, motility, and perinuclear localization were each inhibited; and as a consequence, degradation was also inhibited. This suggested that tubule fission was required for all of the steps of the degradative pathway to follow. The inhibition of acidification observed in dynasore-treated cells may be because the intra-luminal spaces of the endosomes were too large to be acidified, since tubules remained attached to them as a result of fission failure. The dynasore-mediated impairment of endosomal motility may be due to the two motors pulling in opposite directions to produce the force required for fission [36]. The absence of endosomal motility seemed to be responsible for the impaired perinuclear localization observed.

Baf inhibited the endosomal acidification but not motility, while nocodazole inhibited motility but not acidification. In both cases, tubule fission, identified by the segregation of EGF and transferrin, was accomplished even though both perinuclear recruitment and degradation were inhibited. These results imply that endosomal acidification and motility occur independently, but tubule fission is required for both events. Furthermore, both acidification and motility were indispensable for perinuclear localization. The requirement of acidification for perinuclear positioning could be due to the fact that endosomal fusion with perinuclear lysosomes depends on intra-endosomal pH [37].

### Endosomal degradative pathway can be thought of as cascade-regulated

Given all of these results, we propose that the degradative pathway can be considered a cascade with fission at the top of the cascade, followed by endosomal acidification and motility in parallel, followed by juxtanuclear localization, and then degradation at the end (Fig. 5E). Such a cascade is consistent with both our observations here and those in previous reports [15], [16], [17], [18].

Previously, researchers tested the sorting of transferrin and EGF in endosomes following inhibition of sorting nexin-4 (SNX4), a member of a sub-family of sorting nexins that is implicated in endosomal sorting [38]. Following SNX4 inhibition, sorting of transferrin and EGF in the early endosome was inhibited, and transferrin was missorted and destined for degradation. This appears to be in contrast with our model, which proposes that degradation does not occur without removing the recycled molecules. However, since sorting for degradation and recycling are vital for cells, it may be possible that SNX4-depleted cells had adapted to that state, as was observed in dynamin-mutant expressing cells [39].

Since the maturation model was first proposed [40], a number of papers supported the model than another idea that early and late endosomes were stable and separate organelles. Schmid et al., (1988) isolated early and late endosomes, and demonstrated dynamic equilibrium and distinct composition between early and late endosomes, suggesting that recycling components were removed before the transition to late endosomes. But the mechanism underlying the order was still unclear.

This study demonstrated the importance of tubule fission from the early endosome in the degradative pathway and provided new information concerning the degradative pathway. However, there are still many unknowns in this process. First, although the mechanism of tubule fission is beginning to be elucidated [30], [38], [41], it is still largely unknown. Second, while this is the first report that indicates the function of dynamin in the early endosome and in tubule fission, it remains unclear where and how dynamin actually functions. Third, concerning the inhibition of endosomal acidification, it is unknown whether V-ATPase is activated in the absence of fission; namely, it is possible that fission functions as a switch that triggers V-ATPase activation. These unknowns are a good starting point for understanding tubule fission from the early endosome.

## Materials and Methods

### Reagents and antibodies

All chemicals and reagents were of biochemical grade. Mouse anti-EGF antibodies were from R&D Systems, Inc. (Minneapolis, MN). Alexa488-conjugated human transferrin, Alexa555-conjugated EGF and lysotracker DND-26 were from Molecular Probes (Eugene, OR). Mouse anti-EEA1 was from BD transduction Laboratories (Lexington, KY), mouse anti-LAMP1 was from Santa Cruz (Santa Cruz, CA), mouse anti-b-actin was from Sigma-Aldrich (St. Louis, MO), and mouse anti-EGFR was from Cell signaling technology (Danvers, MA). For secondary antibodies, Alexa488-conjugated donkey anti-mouse IgG and rhodamine Red-X–conjugated goat anti-mouse IgG were from Invitrogen. Unlabeled EGF (Roche, Manheim, Germany) was biotinylated using sulfo-NHS-LC-biotin (PIERCE), and excess biotin was removed using Zeba Desalt Spin Columns (PIERCE).

### Cell culture and transfection

HeLa cells were obtained from Cell Resource Center for Biomedical Research, Institute of Development, Aging and Cancer Tohoku University, and maintained in DMEM supplemented with 10% FBS. Transfection was performed with Effectene reagent (QIAGEN, Valencia, CA) according to manufacturer's instructions. The cells were analyzed 24 h after transfection.

### Transport assay

Cells were incubated on ice for 5 min before ligand binding. Alexa488-Tfn (50 µg/ml) and/or alexa555-EGF (20 ng/ml) were bound to cell-surface receptors in DMEM supplemented with 0.1% BSA (serum-free media) on ice for 60 min. After rinsing with ice-cold PBS, the cells were incubated in pre-warmed media at 37°C. After 5 min, the media was changed to pre-warmed media containing DMSO, dynasore (400 µM; ArONIS, Moscow, Russia), bafilomycinA1 (200 nM; Sigma-Aldrich, St. Louis, MO), and nocodazole (10 µM). Cells were further incubated as indicated. For washout, the cells were rinsed once with sufficient media and then further incubated.

### Immunofluorescence

Cells were cultured on coverslips. Following the transport assay, the cells were fixed with methanol for 10 min at −20°C followed by drying at room temperature (RT), or with 3.7% formaldehyde in PBS for 15 min at RT. The cells were then washed three times (5 min for each time) with PBS and then blocked for 30 min at RT with 0.1% BSA in PBS with (for formaldehyde fixation) or without (for methanol fixation) 0.1% TritonX-100. Primary antibodies were diluted in the blocking buffer and incubated with cells for 45 min at RT. After washing three times with PBS, the cells were incubated with secondary antibodies diluted in 1∶200 in 0.1% BSA/PBS for 45 min at RT. After washing three times, the coverslips were mounted on slides using PermaFluor (Thermo, Pittsburgh, PA) and analyzed by spinning disc confocal microscopy (IX-71, Olympus, Tokyo, Japan).

### Electron microscopy

A transport assay was performed using 25 µg/ml peroxydase-conjugated transferrin (Pierce, Rockford, IL), as described above. After rinsing with serum-free media on ice, the cells were incubated for 30 min on ice in DAB, 70 mM NaCl, and 20 mM Hepes, which was supplemented just before use with 0.02% H_2_O_2_. The cells were rinsed three times with PBS on ice and fixed with a buffer containing 2% paraformaldehyde and 2% glutaraldehyde in 0.1 M phosphate buffer (pH 7.4). The cells were observed under a Hitachi H-7100 transmission electron microscope at the central Research Laboratory at Okayama University Medical School, Japan.

### ELISA assay

ELISA plates (Maxisorp, Nunc, Thousand Oaks, CA) were coated overnight with antibodies in 50 mM NaHCO_3_ (pH 9.6) at 4°C. After rinsing twice with PBS, the plates were incubated for 1 h at 37°C in blocking buffer [0.2% BSA, 1% TritonX-100, 0.1% SDS, 1 mM EDTA, 50 mM NaCl, 10 mM Tris-HCl, pH 7.4]. Following the transport assay, the cells were lysed in the blocking buffer and plated on ELISA plates. The plates were incubated for 3 h at 37°C or overnight at 4°C, and then rinsed three times with PBS, incubated for 5 min at RT, then washed three times with PBS. Streptavidin–conjugated horseradish peroxidase (DAKO, Osaka, Japan) in blocking buffer was mounted and incubated for 1 h at RT. The plates were subsequently washed three times with PBS. Horseradish peroxidase was detected using SUMILON (Shizuoka, Japan), and signals were measured using a microplate reader (Hitachi, MTP-300).

### Live-cell imaging and data analysis

Cells were grown in a glass-base dish (IWAKI, Japan), and imaging was performed at 37°C using a stage heater. To visualize the acidification of endosomes, lysotracker (50 nM) was added at 30 min before observation.

For data analysis, colocalization area was measured using MetaMorph software (Universal Imaging Corp., Westchester, PA). Manual tracking was performed using ImageJ software (National Institute of Health). Perinuclear localization was manually judged. “Perinuclear localized” was judged when almost endosomes (∼80%) were localized on perinuclear area, which was defined as internal half area of cytoplasm. Three-dimensional reconstruction was performed by MetaMorph software.

## Supporting Information

Figure S1 **Dynasore induces mitochondria enlargement.** Cells were bound with HRP-transferrin and treated as in **Fig. 1**. After 30 min, the cells were incubated with a DAB-containing solution on ice for 30 min, fixed, and processed for electron microscopy. Scale bar, 500 nm.
(EPS)

Figure S2 **Dynasore inhibits transport from early to late endosomes.** HeLa cells were transfected with GFP-Rab5 (**A-D**) or GFP-Rab7 (**E-H**) and a transport assay was performed with Alexa 555-EGF as in **Figure S1** with (**B**, **D**, **F,** and **H**) or without (**A**, **C**, **E**, and **G**) dynasore. Cells were fixed at 15 min (**A**, **B**, **E**, and **F**) or 30 min (**C**, **D**, **G**, and **H**) post-internalization and mounted.
(EPS)

Figure S3 **Bafilomycin A1 does not effect on the segregation between degradative and recycling pathway.** HeLa cells were internalized with Alexa555-EGF and Alexa488-transferrin for 30 min, fixed and observed by confocal microscopy.
(TIFF)

Movie S1 **Segregation of early endosomes visualized by internalized ligands.** A transport assay was performed using Alexa555-EGF and Alexa488- transferrin in HeLa cells. 10 min after internalization, cells were imaged at 1 frame per 2 seconds for a 2 minute period. The movie is compressed to 7 frames per second.
(MOV)

Movie S2 **Three-dimentional structure of early endosomes in dynasore-treated cells.** Transport assay was performed using Alexa555-EGF and Alexa488-transferrin in HeLa cells, as in **Figure 2**. Cells were fixed, and pictures of different slices were taken within the same cell. Three-dimensional reconstruction was performed using Metamorph software, and the movie was compressed to 7 frames per second.
(MOV)

Movie S3 **Disappearance of endosomal movements in dynasore-treated cells.** A transport assay was performed using Alexa555-EGF and Alexa488-transferrin in dynasore-treated HeLa cells, as in **Figure 3**. Live images were taken just before washout. Frames were captured every 2 sec for a total of 2 min. The movie was compressed to 7 frames per second.
(MOV)

Movie S4 **Reappearance of endosomal movements in cells after washout of dynasore.** A transport assay was performed using Alexa555-EGF and Alexa488-transferrin in dynasore-treated HeLa cells, as in **Figure 3**. Live images were taken 10 min after washout. Frames were captured every 2 sec for a total of 2 min. The movie was compressed to 7 frames per second.
(MOV)

Movie S5 **Endosomal movement under bafilomycin A1 treatment.** A transport assay was performed using Alexa555-EGF in HeLa cells, as in **Figure 1**, with the addition of bafilomycin A1 at 5 min post-internalization. Live images were taken 10 min after internalization. Frames were captured every 2 sec for a total of 2 min. The movie was compressed to 7 frames per second.
(MOV)
