# Supplementary figures and images for: Fission of Tubular Endosomes Triggers Endosomal Acidification and Movement
Source: PLoS One. 2011 May 10;6(5):e19764. doi: 10.1371/journal.pone.0019764 (PMC3091875; doi:10.1371/journal.pone.0019764)

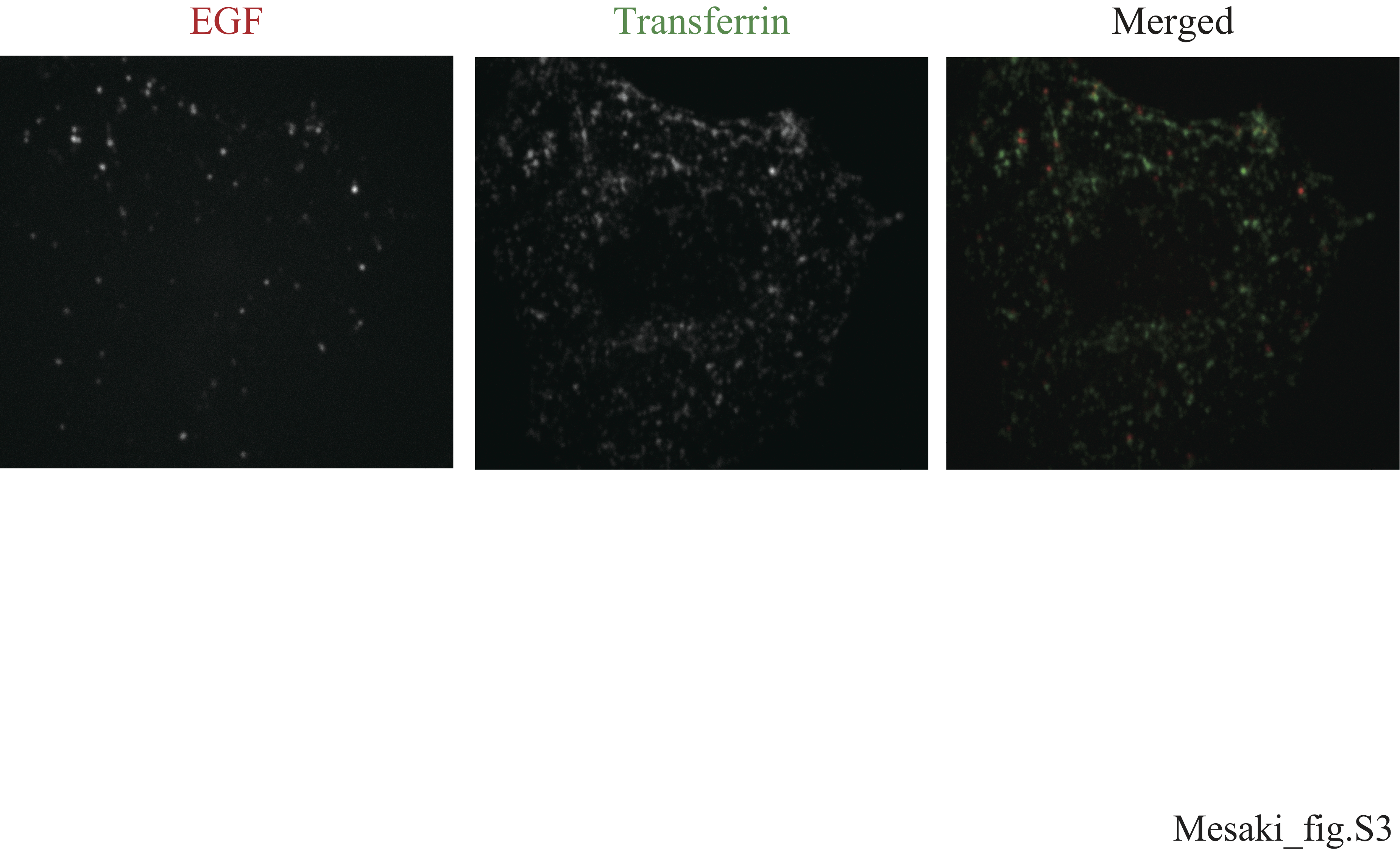

Supplement: Figure S3 — Bafilomycin A1 does not effect on the segregation between degradative and recycling pathway. HeLa cells were internalized with Alexa555-EGF and Alexa488-transferrin for 30 min, fixed and observed by confocal microscopy. (TIFF) [file pone.0019764.s003.tiff]
